# Licochalcone A induces apoptosis in KB human oral cancer cells via a caspase-dependent FasL signaling pathway

**DOI:** 10.3892/or.2013.2929

**Published:** 2013-12-16

**Authors:** JAE-SUNG KIM, MI-RA PARK, SOOK-YOUNG LEE, DO KYOUNG KIM, SUNG-MIN MOON, CHUN SUNG KIM, SEUNG SIK CHO, GOO YOON, HEE-JEONG IM, JAE-SEEK YOU, JI-SU OH, SU-GWAN KIM

**Affiliations:** 1 Regional Innovation Center for Dental Science and Engineering, Chosun University, Gwangju 501-759, Republic of Korea; 2 Department of Oral Physiology, Chosun University, Gwangju 501-759, Republic of Korea; 3 Department of Oral Biochemistry, Chosun University, Gwangju 501-759, Republic of Korea; 4 Department of Oral and Maxillofacial Surgery, Chosun University, Gwangju 501-759, Republic of Korea; 5 Department of Pharmacy, College of Pharmacy, Mokpo National University, Muan, Jeonnam 535-729, Republic of Korea; 6 Department of Biochemistry, Rush University Medical Center, Chicago, IL 60612, USA

**Keywords:** licochalcone A, oral cancer, apoptosis, Fas ligand, caspase

## Abstract

Licochalcone A (Lico-A) is a natural phenol licorice compound with multiple bioactivities, including anti-inflammatory, anti-microbial, anti-fungal and osteogenesis-inducing properties. In the present study, we investigated the Lico-A-induced apoptotic effects and examined the associated apoptosis pathway in KB human oral cancer cells. Lico-A decreased the number of viable KB oral cancer cells. However, Lico-A did not have an effect on primary normal human oral keratinocytes. In addition, the IC_50_ value of Lico-A was determined to be ~50 μM following dose-dependent stimulation. KB oral cancer cells stimulated with Lico-A for 24 h showed chromatin condensation by DAPI staining, genomic DNA fragmentation by agarose gel electrophoresis and a gradually increased apoptotic cell population by FACS analysis. These data suggest that Lico-A induces apoptosis in KB oral cancer cells. Additionally, Lico-A-induced apoptosis in KB oral cancer cells was mediated by the expression of factor associated suicide ligand (FasL) and activated caspase-8 and −3 and poly(ADP-ribose) polymerase (PARP). Furthermore, in the KB oral cancer cells co-stimulation with a caspase inhibitor (Z-VAD-fmk) and Lico-A significantly abolished the apoptotic phenomena. Our findings demonstrated that Lico-A-induced apoptosis in KB oral cancer cells involves the extrinsic apoptotic signaling pathway, which involves a caspase-dependent FasL-mediated death receptor pathway. Our data suggest that Lico-A be developed as a chemotherapeutic agent for the management of oral cancer.

## Introduction

Licochalcone A [Lico-A; (E)-3-[4-hydroxy-2-methoxy-5-(2-methylbut-3-en-2-yl)phenyl]-1-(4-hydroxyphenyl)prop-2-en-1- one] is a natural phenol licorice compound isolated from licorice root (*Radix Glycyrrhizae*). Lico-A has been used for thousands of years as a traditional herbal medicine and is known for its multiple bioactivities including anti-inflammatory (1), anti-microbial (2), anti-malarial (3), and osteogenic activity (4), and anti-angiogenic (5) and anticancer effects (6–9). Moreover, Lico-A has also been suggested to be beneficial for common oro-dental disease via its anti-adhesion properties (10). Although the multiple bioactivities of Lico-A have been revealed in various research fields, its anticancer property in oral cancer is still largely unknown.

Oral cancer is a major worldwide public health issue and may affect any region of the oral cavity, including the lips, tongue, mouth and throat (11,12). Although the pathophysiological studies associated with the development of oral cancer have shown that environmental factors, such as smoking, alcohol and betel quid, may act as critical carcinogens, the etiology of oral cancer is still largely unknown (13–15). However, oral cancer is one of the most prevalent cancers with an incidence rate of 3.9 cases per 100,000 individuals worldwide (16). Even though the clinical treatment for oral cancer has shown significant improvement during the past decade, current clinical treatments consisting of surgery and/or radiotherapy are not totally free from various side-effects, including loss of function and disfigurement. As a result, these side-effects reduce the quality of life of patients with oral cancer. Therefore, various studies are underway to develop effective clinical treatment with minimal side-effects for oral cancer. As part of these studies, intensive research is being carried out to develop novel chemotherapeutic agents from edible herbal plants or natural products (17–21).

Apoptosis is generally defined as programmed cell death via a precisely regulated cell suicide process, which is mediated by different intracellular and extracellular biological mechanisms (22). Apoptosis largely occurs through two pathways; one is the mitochondrial apoptotic pathway and the second is the death receptor pathway. The mitochondrial pathway, known as intrinsic apoptotic signaling, is triggered by the release of cytochrome *c* due to the loss of mitochondrial transmembrane potential (23,24). The death receptor pathway, known as extrinsic apoptotic signaling, is mediated by sequential activation of caspase-8 and −3 and poly(ADP-ribose) polymerase (PARP), after interaction with death receptor and its ligands, such as TRAIL and factor associated suicide ligand (FasL) (25). Importantly, apoptosis has emerged as an important mechanism for the anticancer effects of chemotherapeutic agents developed from herbal plants.

Hence, the aim of the present study was to determine whether Lico-A has potential to function as a chemotherapeutic agent for the treatment of KB oral cancer cells without affecting normal cells originating from the oral cavity. Furthermore, the present study aimed to evaluate the potential apoptotic effect of Lico-A and to elucidate the Lico-A-induced apoptotic signaling pathway in KB oral cancer cells.

## Materials and methods

### Cell culture

Normal human oral keratinocytes (NHOKs) were purchased from ScienCell Research Laboratories (Carlsbad, CA, USA). The NHOKs were maintained in DMEM (Gibco, Grand Island, NY, USA) containing 10% fetal bovine serum (FBS) (Invitrogen, Carlsbad, CA, USA) at 37°C in an atmosphere containing 5% CO_2_. The human oral squamous cell carcinoma cell line, KB, was obtained from the American Type Culture Collection (ATCC; Manassas, VA, USA) and cultured according to the cell culture instructions provided. Briefly, KB cells were grown in MEM containing 10% FBS at 37°C in an atmosphere containing 5% CO_2_.

### Cell viability assay

Both KB oral cancer cells and NHOKs were seeded at a density of 5×10^5^ cells/well in 96-well plates and allowed to attach to the well overnight. After incubation, cultured cells were stimulated with various concentrations of Lico-A in triplicate and incubated at 37°C in a 5% humidified CO_2_ incubator for 24 h. Subsequently, 3-(4,5-dimethylthiazol-2-yl)-2,5-diphenyltetrazolium bromide (MTT) was added to each well, and incubation was continued for a further 4 h at 37°C. To dissolve the formazan formed from MTT, the cells were resuspended in 200 μl dimethyl sulfoxide (DMSO), and the optical density (OD) of the solution was determined using a spectrometer at a wavelength of 570 nm. The experiments were repeated 3 times, independently. The mean optical density (OD) ± SD for each group of replicates was calculated. The entire procedure was repeated 3 times. The inhibitory rate of cell growth was calculated using the equation: $\%\;\text{Growth inhibition}=(1-{\text{OD}}_{\text{extract treated}})/{\text{OD}}_{\text{negative control}}\times 100$.

### Live/dead cell viability assay

The live/dead cell viability assay was carried out as previously described (26), using calcein AM to stain the live cells and ethidium bromide homodimer 1 to stain the dead cells. These reagents were obtained from Molecular Probes (Eugene, OR, USA). For the cell survival assay, KB oral cancer cells and HNOKs were plated in a chamber slide, stimulated with berberine for 24 h and stained with green calcein AM and ethidium bromide homodimer 1 according to the manufacturer's protocol. The cells were then observed and photographed by inverted phase-contrast microscopy.

### DNA fragmentation assay

KB oral cancer cells were collected after stimulation with Lico-A (0, 25 and 50 μM) for 24 h and were rinsed 3 times in phosphate-buffered saline (PBS) at 4°C. This was followed by degradation using 100 μl cell lysate buffer (1% NP-40, 20 mM EDTA, 50 mM Tris-HCl, pH 7.5) at 4°C for 10 min, followed by centrifugation at 12,000 × g for 30 min. RNase A was added to the supernatant and incubated at 37°C for 1 h. Proteinase K was then added to the supernatant, and incubation was conducted at 37°C for 8 h. An equal volume of isopropanol was then added and kept at −80°C for 24 h to precipitate the genomic DNA. The supernatant was removed after centrifugation at 12,000 × g for 15 min at 4°C. The supernatant was allowed to dry naturally and was dissolved in TE buffer, followed by electrophoresis on 1.5% agarose gels. A gel imaging system was used for observation and capturing images.

### 4′,6-Diamidino-2-phenylindole (DAPI) staining

KB oral cancer cells stimulated with 25 and 50 μM Lico-A for 24 h were fixed with 4% paraformaldehyde prior to washing with PBS. The washed cells were then stained with 1 mg/ml 4′,6′-diamidino-2-phenylindole dihydrochloride (DAPI) (Roche Diagnostics) for 20 min in the dark. The DAPI-stained images were capturing using fluorescence microscopy (Eclipse TE200; Nikon Instruments, Melville, NY, USA).

### Caspase-3/−7 activity assay

The apoptosis executioner caspase-3/−7 activity was determined using the cell-permeable fluorogenic substrate, PhiPhiLux-G_1_D_2_ (Oncoimmunin Inc., Gaithersburg, MD, USA), according to the manufacturer's instructions.

### Annexin V-FITC and propidium iodide (PI) staining and flow cytometric analysis

Apoptosis was determined by Annexin V-fluorescein isothiocyanate assay. Cells were washed in PBS twice and resuspended in binding buffer (BD Biosciences, San Diego, CA, USA). Annexin V-fluorescein isothiocyanate and 7-amino-actinomycin D (BD Biosciences) were added to the cells, which were then incubated in the dark for 15 min. Cells were then added and resuspended in 400 ml of binding buffer. Cells were analyzed using a fluorescence activated cell sorting FACSCalibur flow cytometer (Becton-Dickinson, San Jose, CA, USA). Data analysis was performed using standard CellQuest software (Becton-Dickinson).

### RNA isolation and quantitative PCR analysis

Total RNA was isolated using TRIzol reagent (Life Technologies, Carlsbad, CA, USA), according to the manufacturer's instructions. Total RNA (1 μg) was reverse transcribed into first strand cDNA using ThermoScript™ RT-PCR system (Life Technologies). For quantitative PCR (qPCR), cDNA was amplified using a SureCycler 8800 (Agilent Technologies, Santa Clara, CA, USA) and 2X TOPsimple™ DyeMIX-nTaq (Enzynomics, Seoul, Korea), according to the manufacturer's instructions. Gene expression was determined using agarose gel electrophoresis. GAPDH was used as the internal controls in the reactions for normalization. Primer used for qPCR were as follows: for caspase-3, forward primer, 5′-TCTTGG CGAAATTCAAAGGATGGC-3′ and reverse primer, 5′-TT TGTGAGCATGGAAACAATACATGG-3′; for caspase-3, forward primer, 5′-GGCTTTGACCACGACCTTTGAAGA-3′ and reverse primer, 5′-GGAAGGGCACTTCAAACCAGTG AA-3′; for FasL, forward primer, 5′-GCTGGAGTCATGACA CTAAGTCAA-3′ and reverse primer, 5′-CTCTGCAAGAGT ACAAAGATTGGC-3′; for GAPDH, forward primer, 5′-AGCC TCAAGATCATCAGCAATG-3′ and reverse primer, 5′-ATG GACTGTGGTCATGAGTCCTT-3′.

### Immunoblotting

Cell and tissue lysates were prepared using modified radioimmunoprecipitation assay buffer (1 M Tris-HCl, 150 mM NaCl, 1% Triton X-100, 2 mM EDTA) with protease inhibitor and phosphatase inhibitor cocktail (both from Sigma, USA). Total protein concentrations of the cell lysates were determined by bicinchoninic acid protein assays (Pierce). Equal amounts of protein were resolved by 10% sodium dodecyl sulfate-polyacrylamide gel electrophoresis and transferred to nitrocellulose membranes for immunoblotting analyses. After blocking with 5% bovine serum albumin (BSA) in TBS-T at room temperature for 1 h, the membranes were sequentially blotted with primary antibodies at 4°C overnight. After rinsing in TBS-T, the membranes were incubated with HRP-conjugated secondary antibody at room temperature for 1 h. Immunoreactivity was visualized using the ECL system (Amersham Biosciences).

### Statistical analysis

Data are expressed as the means ± SD of 3 individual experiments performed in triplicate. Statistical analysis was performed using the Student's t-test, and p<0.05 was considered to indicate a statistically significant result.

## Results

### Lico-A suppresses the viability of KB oral cancer cells, but does not affect the viability of NHOKs

**Figure 1:**
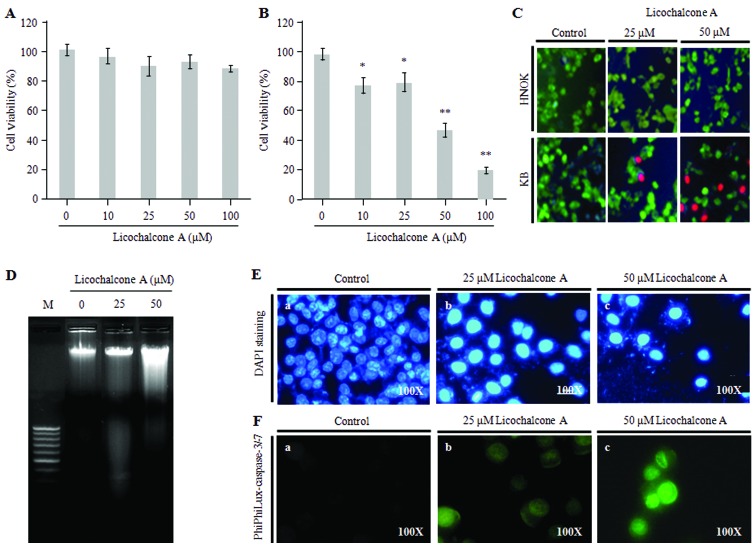
Licochalcone A selectively induces the apoptosis of human KB oral cancer calls, but not human normal oral keratinocytes. (A) Human normal oral keratinocytes and (B) KB oral cancer cells were stimulated with different doses of licochalcone A (0, 20, 25, 50 and 100 μM) for 24 h. After the indicated stimulating condition, cell viability was measured using an MTT assay, and cell viability was quantified by measuring the optical density at 570 nm. The data represent triplicate results of 3 independent experiments and are expressed as means ± SD (^*^p<0.05 and ^**^p<0.01 as compared to the control). (C) Cell survival assay using green calcein AM for live cells (green color) and ethidium bromide homodimer 1 for dead cell (red color). (D) For analysis of genomic DNA fragmentation, KB cells were stimulated with different doses of licochalcone A for 24 h, harvested, and the genomic DNA was isolated. Genomic DNA isolated from the KB cells was electrophoresed on 1.5% agarose gel containing ethidium bromide and visualized using UV. (E) DAPI staining to identify nuclear condensation. KB cells were stimulated with different doses of licochalcone A for 24 h, fixed with 4% paraformaldehyde and nuclear staining was performed using DAPI. The cells stained by DAPI were observed under a fluorescence microscope. (F) Caspase-3 intracellular activity assay. KB cells were stimulated with different doses of licochalcone A for 24 h, fixed with 4% paraformaldehyde and nuclear staining was performed using PhiPhiLux-caspase-3/−7. The cells stained with DAPI were observed under a fluorescence microscope. DAPI, 4′,6-diamidino-2-phenylindole.

The effect of Lico-A on the viability of KB oral cancer cells and NHKOs was assessed by the MTT assay. As shown in Fig. 1A, Lico-A did not have an effect on the NHKOs, which are primary normal oral keratinocytes originating from the human oral cavity, when compared to the control. In contrast, KB oral cancer cells stimulated with different concentrations of Lico-A for 24 h exhibited-significantly decreased cell viability when compared to the control (Fig. 1B). Particularly, 50 and 100 μM Lico-A decreased the cell viability by ~50 and 80% when compared with the control. These data indicate that the IC_50_ value of Lico-A in KB oral cancer cells was ~50 μM.

### Lico-A induces apoptotic phenomena, such as DNA fragmentation, chromatin condensation and activation of caspase-3, in KB oral cancer cells

Following stimulation of KB oral cancer cells with Lico-A for 24 h, morphological changes, such as shrinkage, aggregation and the increase in detachment of cells from the surface of culture vessels, were observed using microscopy. As shown in Fig. 1D, genomic DNA isolated from KB oral cancer cells stimulated with Lico-A clearly showed the formation of DNA trailing when compared to the control. These data indicate that DNA breakage or fragmentation, which only occurs when cell apoptosis takes place, was induced by Lico-A. To observe the morphological nuclear change in KB oral cancer cells stimulated with Lico-A, we performed nuclear staining using DAPI. As shown in Fig. 1E, the population of KB oral cancer cells that attached to the surface of the culture vessel was significantly decreased after Lico-A stimulation. Furthermore, the number of condensed and fragmented nuclei was significantly increased in KB oral cancer cells following stimulation with Lico-A (Fig. 1E). Furthermore, to confirm the Lico-A-induced apoptosis, caspase-3 intracellular activity assay was performed using capsase-3/−7 PhiPhiLux staining. As shown in Fig. 1F, activated caspase-3 was significantly detected in the cytosol of KB oral cancer cells stimulated with Lico-A for 24 h in a dose-dependent manner. In contrast, in KB oral cancer cells stimulated without Lico-A as the control activated caspase-3 was not observed. These data suggest that Lico-A-induced cell death was mediated by apoptosis.

### Lico-A increases the number of apoptotic cells in a time-dependent manner

**Figure 2:**
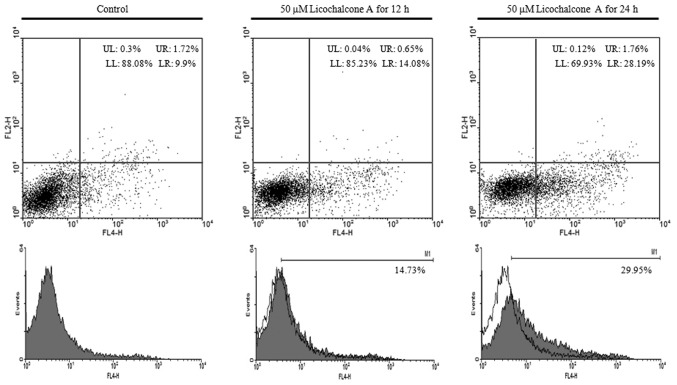
Licochalcone A treatment increases the apoptotic population in KB oral cancer cells. To assess the licochalcone A-induced KB oral cancer cell apoptosis, FACS analysis was performed by Annexin V and PI staining. KB oral cancer cells were cultured in complete medium for 24 h and stimulated with 50 μM licochalcone A (IC_50_ concentration) for 12 and 24 h. After stimulation, the cells were analyzed by flow cytometry.

To determine whether Lico-A-induced cell death is associated with the induction of apoptosis, KB oral cancer cells were stimulated with 50 μM Lico-A for 12 and 24 h and subsequently co-stained with the apoptotic markers Annexin V-FITC and necrotic marker PI. As shown in Fig. 2, the number of apoptotic cells at the early stage of apoptosis was gradually increased by 14.08 and 28.19% at 12 and 24 h, respectively, in a time-dependent manner. In addition, as shown in Fig. 2, the percentage of Annexin V-FITC-positive cells at both the early and late stage of apoptosis was gradually increased up to 29.95% at 24 h when compared with the control.

### Lico-A-induced apoptosis is mediated via the FasL/PARP axis

**Figure 3:**
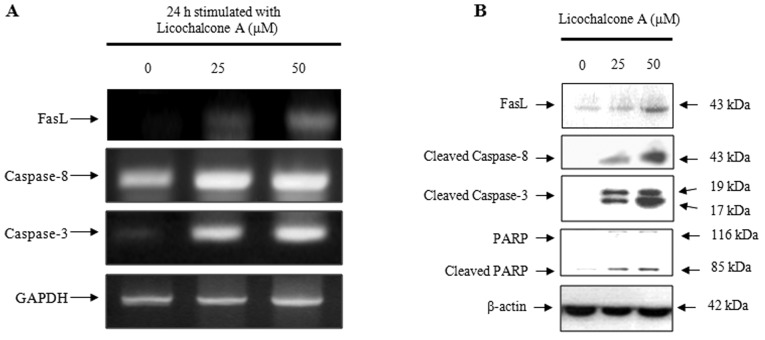
Licochalcone A-induced apoptosis in KB oral cancer cells is mediated by expression and activation of caspases. (A) Total RNA from KB oral cancer cells stimulated with licochalcone A for 24 h was isolated using TRIzol reagent. cDNA synthesis was carried out with 1 μg total RNA using reverse transcriptase. qPCR was performed using synthesized cDNA. The amplified PCR product was then electrophoresed on 1% agarose gel. (B) Licochalcone A activated the extrinsic apoptosis signaling pathway in KB oral cancer cells. KB oral cancer cells were stimulated with 25 and 50 μM licochalcone A for 24 h, harvested and lysed using cell lysate buffer. Protein quantification and western blotting were performed. qPCR, quantitative PCR.

To verify the mechanism by which Lico-A induces the apoptosis of KB oral cancer cells, qPCR and immunoblotting were performed to measure the expression of apoptosis-related genes at both the mRNA and protein levels. As shown in Fig. 3A, FasL, which is an apoptotic ligand and triggers the extrinsic apoptotic pathway, was significantly induced by Lico-A in KB oral cancer cells. Subsequently, caspase-8 and −3, which are downstream targets of FasL, were significantly induced by Lico-A at the mRNA level. However, apoptosis requires the activation of the caspase cascade and PARP. As shown in Fig. 3B, FasL was significantly expressed in KB cells following Lico-A treatment in a dose-dependent manner. Sequentially, upregulated FasL by LicoA triggered the cleavage of pro-caspase-8 into cleaved (activated) caspase-8 (43 kDa). In the next step, cleaved caspase-8 induced the cleavage of pro-caspase-3 as its downstream target. Activated caspase-3 (17 and 19 kDa) gradually increased in a dose-dependent manner. Furthermore, cleaved PARP (85 and 25 kDa) was increased by activated caspase-3 when compared with the control. β-actin was used as an internal control for normalization. Therefore, these data clearly indicate that Lico-A-induced apoptosis in KB oral cancer cell was mediated by the extrinsic apoptotic pathway via the FasL/PARP axis.

### Lico-A-induced apoptosis is regulated by the activation of caspases in KB oral cancer cells

**Figure 4:**
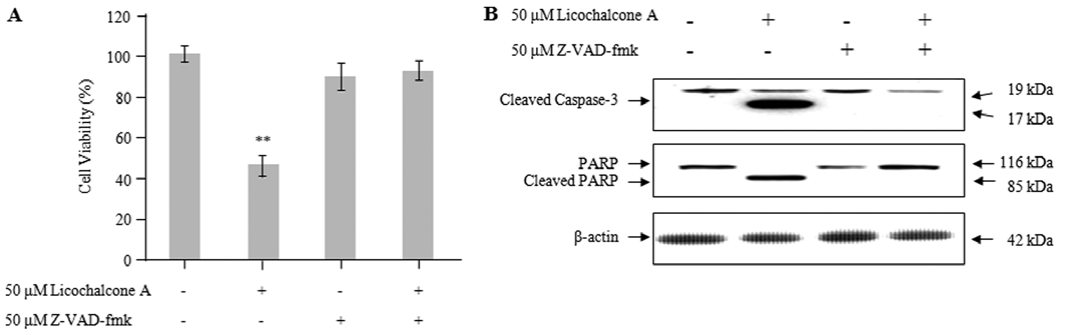
Licochalcone A-induced apoptosis in KB oral cancer cells is mediated by caspase activation. (A) Z-VAD-fmk, a caspase inhibitor, inhibited licochalcone A-induced KB cell apoptosis. KB cells were stimulated with 50 μM licochalcone A present with/without 50 μM Z-VAD-fmk for 24 h. After stimulation, cell viability was assessed by MTT assay. (B) The increased expression and activation of apoptosis-related proteins by licochalcone A in KB oral cancer cells were suppressed by Z-VAD-fmk. KB cells were stimulated with 50 μM licochalcone A present with/without 50 μM Z-VAD-fmk for 24 h, harvested, and lysed using cell lysate buffer. Protein quantification and immunoblotting were performed.

Apoptotic signals are mediated by the activation of the caspase cascade, which is a key hallmark of apoptosis. We, therefore, determined a direct involvement of caspase activation in Lico-A-induced KB oral cancer cell apoptosis. As shown in Fig. 4A, 50 μM of Z-VAD-fmk, a pan-caspase inhibitor, had no effect on the cell viability in KB oral cancer cells similar to the non-treated control. In addition, 50 μM Lico-A consistently decreased the cell viability by ~50% (Fig. 1B). However, Lico-A-induced cell cytotoxicity in KB oral cancer cells was partially recovered by treatment with Z-VAD-fmk as compared with Lico-A stimulation only. Furthermore, Z-VAD-fmk treatment led to inhibition of Lico-A-induced caspase-3 and PARP activation (Fig. 4B). These data indicate that the Lico-A-induced apoptosis was regulated by the activation of the caspase cascade in KB oral cancer cells.

### ERK^MAPK^ and p38^MAPK^ pathways are required for Lico-A-induced FasL upregulation and subsequent apoptosis in KB oral cancer cells

**Figure 5:**
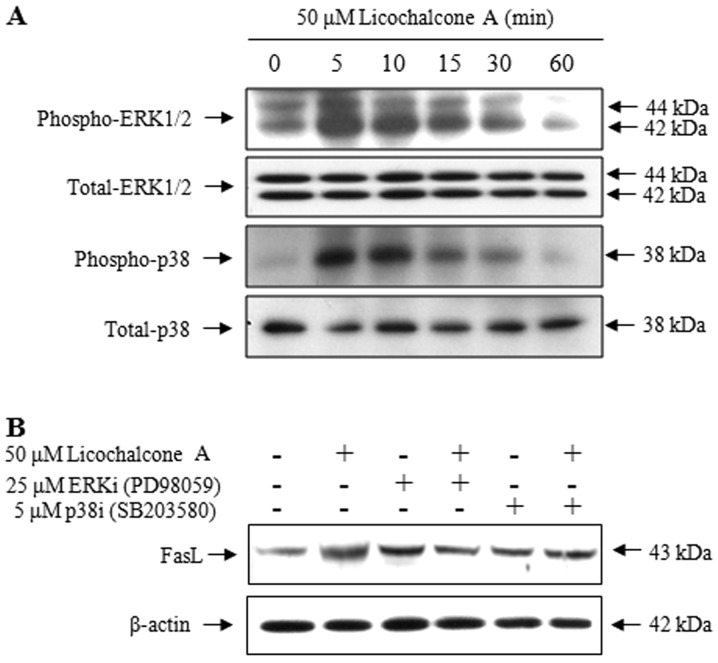
Licochalcone A-induced apoptosis in KB oral cancer cells is associated with activation of the MAPK signaling pathway. (A) Serum-starved KB oral cancer cells were stimulated with 50 μM licochalcone A for the indicated periods of time. Cell lysates were then prepared and analyzed by immunoblotting with specific anti-phospho-ERK, anti-total-ERK, anti-phospho-p38 and anti-total p38 antibodies. (B) Serum starved KB oral cancer cells were treated with 50 μM licochalcone A in the presence or absence of the ERK^MAPK^ pathway-specific inhibitor PD98059 (ERKi; 25 μM) and the p38^MAPK^ pathway-specific inhibitor SB203580 (p38i; 5 μM). The cells were harvested 24 h after the initiation of each treatment to perform immunoblotting for Fas. β-actin was used as a control for normalization.

To elucidate the apoptotic signaling pathways involved in the Lico-A-induced FasL expression in KB oral cancer cells, we examined the phosphorylation of MAPK signaling pathways that have previously been linked to FasL expression in the apoptosis of cancer cells. As shown in Fig. 5A, 50 μM Lico-A activated both ERK and p38, as reflected by phosphorylation within 5 min and sustained activation for 30 min after stimulation with Lico-A. There was no significant activation of JNK^MAPK^ by Lico-A in KB oral cancer cells. To determine which pathway was involved in Lico-A-induced FasL expression, we performed immunoblotting in the presence or absence of pathway-specific inhibitors of either ERK (PD98059) or p38 (SB203580). The presence of each inhibitor suppressed the Lico-A-mediated FasL expression in KB oral cancer cells (Fig. 5B). Collectively, these findings demonstrated that Lico-A-induced apoptosis of KB oral cancer cells was mediated by the upregulation of FasL via the activation of both the ERK^MAPK^ and p38^MAPK^ signaling pathways.

## Discussion

Oral cancers are common malignancies which have emerged as major international health issues. Global cancer statistics has revealed that the annual incidence of oral cancer exceeds 270,000 cases worldwide (27). Although the clinical treatment for oral cancer has improved, the 5-year survival rate for patients with oral cancer is approximately 50% (28). Even though chemotherapy is one of the important therapeutic strategies for oral cancer, it is still limited by various side-effects such as high toxicity and drug tolerance. Therefore, there is an urgent demand for the development of effective clinical drugs with fewer side-effects. Based on these requirements, natural therapies, which use the natural compounds derived from medicinal plants and traditional Oriental medicine, are being developed to overcome the side-effects of chemotherapeutic reagents.

*Glycyrrhiza uralensis* Fischer is one of the representative medicinal herbal plants for the treatment of sore throat, cough, bronchitis, peptic ulcers, arthritis and allergic disease in traditional Oriental medicine (29,30). In addition, Lico-A, the major bioactive compound isolated from *Glycyrrhiza* sp., has been reported to have various biological activities such as anti-inflammatory (31,32), anti-microbial (33), anti-angiogenic (34), anti-obesity (35) and osteogenic effects (4). In the present study, we demonstrated that Lico-A suppressed the proliferation and induced the apoptosis of KB oral cancer cells via death receptor-mediated caspase activation.

First, we assessed the cell cytotoxicity of Lico-A in both human KB oral cancer cells and primary human oral normal keratinocytes to determine the possibility of its use as a potential chemotherapeutic agent for treating oral cancer. As shown in Fig. 1, the various concentrations of Lico-A did not affect the cell viability in primary human normal oral keratinocytes. In contrast, cell cytotoxicity was significantly increased in human KB oral cancer cells stimulated with Lico-A in a dose-dependent manner. Notably, the cell viability of KB cells was effectively decreased by ~50% at the concentration of 50 μM Lico-A for 24 h. Xiao *et al* also reported that the cell cytotoxicity of gastric cancer cells including MKN-28, AGS and MKN-45 was significantly increased following treatment with ~50 μM of Lico-A for 24 h (8). In contrast, Lico-A exhibited less cytotoxicity to normal human gastric mucosal cells similar to our demonstrated results. These data clearly suggest that Lico-A enhanced the cancer cell-specific cytotoxicity without affecting normal cells. Next, to investigate the mechanism by which Lico-A induces human KB oral cancer cell death, we analyzed DNA fragmentation, morphological alteration and caspase-3 expression following Lico-A stimulation. As shown in Fig. 1D, Lico-A significantly induced DNA fragmentation in human KB oral cancer cells dose-dependently. Furthermore, the morphological alterations including chromatin condensation and a decrease in the cell population were observed in human KB oral cancer cells stained with DAPI after Lico-A treatment (Fig. 1E). As a key feature of apoptotic cell death, both DNA fragmentation and chromatin condensation were observed in human KB oral cancer cells stimulated with Lico-A. During apoptotic events, DNA fragmentation is mediated by caspase-activated DNase, which is an endonuclease found in the extrinsic apoptotic pathway and is activated by caspase-3 (36). Therefore, we performed the caspase-3 intracellular activity assay using PhiPhiLux-caspase-3 to detect the activated caspase-3 in human KB oral cancer cells stimulated with Lico-A. As shown in Fig. 1F, activated caspase-3 was significantly detected intracellularly in human KB oral cancer cells stimulated with Lico-A. In addition, the apoptotic population at both the early and late stages of apoptosis was significantly increased in human KB oral cancer cells stimulated with 50 μM of Lico-A in a time-dependent manner (Fig. 2). These data indicate that Lico-A significantly induced the activation of caspase-3. Furthermore, activated caspase-3 induced the cleavage of the inhibitor of caspase-activated DNase for formatting the caspase-activated DNase. Subsequently, caspase-activated DNase fragmented the genomic DNA of human KB oral cancer cells stimulated with Lico-A. However, these data are consistent indicating that Lico-A-induced human KB oral cancer cell death is closely associated with apoptosis via activation of caspases.

The factor associated suicide ligand (FasL), an important regulatory factor of apoptosis, initiates the death receptor-mediated extrinsic apoptotic pathway through the activation of caspase-8 and −3 and PARP, sequentially, after binding with receptor FasR spanned on the surface of target cells (37,38). As shown in Fig. 3, the expression of FasL was significantly upregulated by Lico-A in the KB oral cancer cells. Subsequently, upregulated FasL triggered a caspase cascade and subsequently resulted in the activation of apoptotic factors, including caspase-8 and −3. Finally, activated caspase-3 cleaved its major substrate PARP resulting in consequent apoptosis. These data clearly suggest that caspase-3 is activated in response to Lico-A in KB oral cancer cells. Therefore, to further evaluate the role of caspase-3 in Lico-A-induced apoptosis, KB oral cancer cells were stimulated with Z-VAD-fmk, a specific caspase-3 inhibitor, to suppress the cleavage of caspase-3. As shown in Fig. 4A, Z-VAD-fmk significantly inhibited the Lico-A-induced apoptosis of KB oral cancer cells. Furthermore, the activation of caspase-3 and its major substrate PARP in KB oral cancer cells stimulated with Lico-A was significantly suppressed by Z-VAD-fmk, indicating that Lico-A-induced apoptosis was dependent on caspase-3 activity (Fig. 4B).

To further investigate the signaling pathway involved in Lico-A-induced apoptosis in KB oral cancer cells, we assessed the effect of Lico-A on mitogen-activating protein kinases, which are associated with the apoptotic signaling pathway. As shown in Fig. 5A, Lico-A induced the phosphorylation of ERK and p38 in a time-dependent manner. However, we did not detect any significant phosphorylation of JNK following Lico-A treatment in KB oral cancer cells. Therefore, to confirm the role of ERK and p38 inhibition in Lico-A-induced FasL expression, KB oral cancer cells were pre-stimulated with pharmacological inhibitors (PD98059 for ERK and SB203580 for p38) for 2 h to turn off each MAPK signaling and then Lico-A was administered for 24 h. Lico-A-induced FasL expression was significantly suppressed by the inhibition of ERK and p38 signaling (Fig. 5B). These results indicate that ERK and p38 are required for the Lico-A-induced FasL expression and apoptosis in KB oral cancer cells. Binding of FasL to its receptor FasR has been shown to activate MAPK, and its activation is required for apoptosis of human hepatocellular carcinoma Huh7 cells (39). Therefore, we demonstrated that Lico-A induced the extrinsic apoptotic signaling pathway in KB oral cancer cells via the upregulation of FasL through both ERK and p38 activation. In conclusion, Lico-A may be developed as a chemotherapeutic agent for the management of oral cancer.
